# Dataset for electronic payment performance in Nigerian banking system: A trend analysis from 2012 to 2017

**DOI:** 10.1016/j.dib.2018.07.046

**Published:** 2018-07-27

**Authors:** Oludare Samuel Fadoju, Grace Evbuomwan, Felicia Olokoyo, Oyeladun Oyedele, Olurotimi Ogunwale, Oladayo Oluremi Kolawole

**Affiliations:** Department of Banking and Finance, Covenant University, Canaanland KM 10, Idiroko Road, Ota, Nigeria

**Keywords:** Adoption, Competitiveness, Digitalization, Electronic banking, E-payment performance

## Abstract

The advent of Information and Communications Technology (ICT) in the new age has led to the digitalization of business processes including banking. For performance measurement among other usefulness, the dataset for these adopted electronic banking channels – Automated Teller Machines, Internet (Web) Transactions, Mobile Payments, Instant Payments, Electronic Fund Transfer, Point of Sales (POS), Automated Cheque Clearing and e-BillsPay was sourced. This dataset gives a trend analysis of e-payment performance of transactions both in value and volumes on each channel as consummated on the platform of Nigeria Inter-Bank Settlement System (NIBSS) in the last six years covering 2012–2017.

## Specifications Table

**Table:** 

Subject area	Banking
More specific subject area	Electronic Banking
Type of data	Table and Graph
How data was acquired	Downloaded from www.cbn.gov.ng, www.nibss-plc.com.ng
Data format	Raw
Experimental factors	Performance Review of e-payment channels of Nigerian Banks
Experimental features	Data in actual transaction values and volumes
Data source location	Central Bank of Nigeria, Nigeria Inter-Bank Settlement System-Lagos, Nigeria
Data accessibility	Data is with this article and available from www.cbn.gov.ng, www.nibss-plc.com.ng
Related research article	

## Value of the data

• The dataset sheds light on the post-adoption and performance of electronic banking in Nigeria which is one of the largest economy in the Africa continent.
• The dataset is useful for research work on determining the responsiveness of bank customers to e-banking products, e-payment fraud and cashless policy agenda of regulatory authorities.
• The dataset is valuable for further guidance to researchers who act as consultants on policy formulation, financial advisory services and performance measurement.
• The dataset is valuable to manufacturers in ICT industry producing e-products equipment or gadgets such as Cards, Point of Sales (POS) machines etc. in analyzing market opportunities and production focus.
• The dataset can be used by research and development units of Mobile telecommunications operators, Internet Service Providers (ISPs) and Financial Technology (FinTech) companies for market analysis, forecasting and opportunities that lies ahead.

## 1 Data

The dataset represents actual e-payment transactions both in volume and values consummated by various individuals and corporate customers of Nigeria banks nationwide in the last six years from January 2012 to December 2017. This dataset gives a breakdown of transactions both in volume and values for eight different e-payment channels authorized and adopted for settlement by all banks in Nigeria. Data was derived from the repositories of Nigeria Inter-Bank Settlement System (NIBSS) and the Central Bank of Nigeria (CBN). The dataset has been analyzed using table and pictorial presentations.

**Fig. 1.1:**
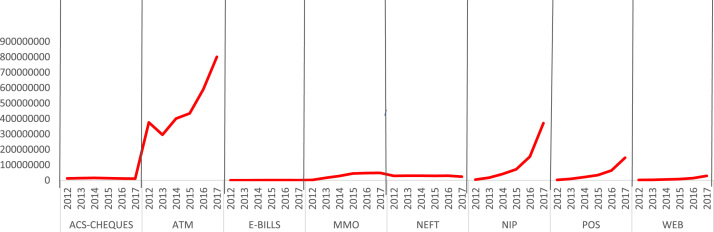
Volume of e-payment channels.

In Fig. 1.1 dataset shows the total volume of transactions carried out on each of the e-payment channels from 2012 to 2017 by all the banks in Nigeria – Automated Clearing System (ACS) Cheques – 77,652,000, NIBSS Electronic Fund Transfer (NEFT) – 170,868,138, Automated Teller Machine (ATM) – 2,895,863,700, Point of Sales (POS) – 276,531,743, Internet (Web) – 61,803,981, Mobile Money Operations (MMO) – 184,759,280, NIBSS Instant Payment (NIP) – 658,102,383, e-BillsPay – 3,727,526.

**Fig. 2.1:**
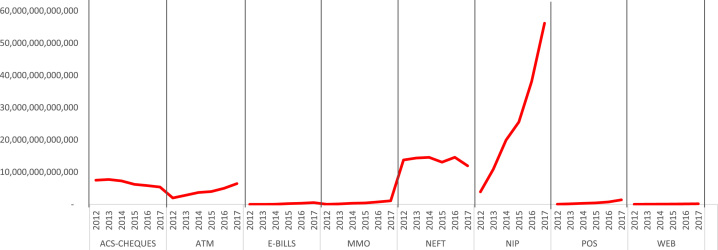
Value of e-payment channels.

Fig. 2.1 depicts the total Naira value from 2012 to 2017 – ACS Cheques valued at N39.9 Trillion, NEFT – N82.3 Trillion, ATM – N23.9 Trillion, POS – N3.1 Trillion, Web – N561.6 Billion, MMO – N2.8 Trillion, NIP – N154.5 Trillion and e-BillsPay – N1.1 Trillion.

**Fig. 3.1:**
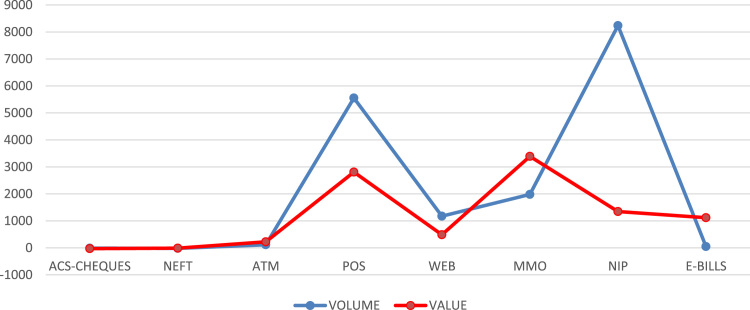
Percentage growth of e-payment channels.

Fig. 3.1 depicts the growth rate in both volume and value of each e-payment channels over the six year period. ACS Cheques has -11.1% and -28.1%, NEFT has -18.1% and -13.1%, ATM has 113.2% and 224.5%, POS has 5552.8% and 2809.1%, Web – 1173.5% and 484.8%, MMO has 1980.4% and 3391%, NIP has 8234.8% and 1343.8% while e-BillsPay has 49.8% and 1116.6%.

This dataset is useful for defining the existing relationship among e-payment channels in mathematical form for further research work.

**Fig. 4.1:**
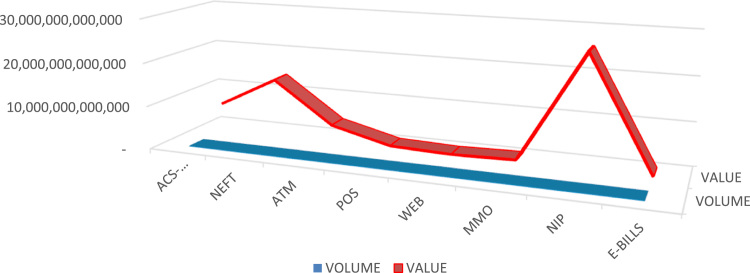
Annual average for e-payment channels.

Fig. 4.1 depicts annual average in both volume and value for each e-payment channels. For the six years, ACS Cheque – 12,942,000 and N6.6 Trillion, NEFT – 28,478,023 and N13.7 Trillion, ATM – 482,613,950 and N3.98 Trillion, POS – 46,088,624 and N523.1 Billion, Web – 10,300,664 and N93.6 Billion, MMO – 30,793,213 and N468.9 Billion, NIP – 109,683,731 and N25.7 Trillion while e-BillsPay has 621.254 and N192.5Billion.

## 2 Experimental design, materials, and methods

The dataset is a raw data for a six year period starting from 2012 to 2017 pooled from the online repositories of the apex regulatory body, the Central Bank of Nigeria (CBN) and the Nigeria Inter-Bank Settlement System (NIBSS). The Nigeria banking system has undergone series of reform agenda like consolidation, universal banking and cashless policy but had stability and confidence restored within the last six years hence the choice of dataset collection period. The dataset was analyzed using Microsoft excel applying sum total and simple average formula. Since NIBSS is jointly owned by all the listed banks and CBN, the dataset gives a good degree of reliability level for the analysis. Details of similar research work that have analyzed dataset in a descriptive form are [1], [2], [3], [4], [5], [6], [7], [8], [9].
